# Integrating regulatory surveys and citizen science to map outbreaks of forest diseases: acute oak decline in England and Wales

**DOI:** 10.1098/rspb.2017.0547

**Published:** 2017-07-19

**Authors:** Nathan Brown, Frank van den Bosch, Stephen Parnell, Sandra Denman

**Affiliations:** 1 Computational and Systems Biology, Rothamsted Research, Harpenden, UK; 2 School of Environment and Life Sciences, University of Salford, Peel Building, Manchester, Lancashire, UK; 3 Forest Research, Farnham, Surrey, UK

**Keywords:** citizen science, disease survey, dispersal, acute oak decline

## Abstract

The number of emerging tree diseases has increased rapidly in recent times, with severe environmental and economic consequences. Systematic regulatory surveys to detect and establish the distribution of pests are crucial for successful management efforts, but resource-intensive and costly. Volunteers who identify potential invasive species can form an important early warning network in tree health; however, what these data can tell us and how they can be best used to inform and direct official survey effort is not clear. Here, we use an extensive dataset on acute oak decline (AOD) as an opportunity to ask how verified data received from the public can be used. Information on the distribution of AOD was available as (i) systematic regulatory surveys conducted throughout England and Wales, and (ii) ad hoc sightings reported by landowners, land managers and members of the public (i.e. ‘self-reported’ cases). By using the available self-reported cases at the design stage, the systematic survey could focus on defining the boundaries of the affected area. This maximized the use of available resources and highlights the benefits to be gained by developing strategies to enhance volunteer efforts in future programmes.

## 1. Introduction

The threat to natural environments and commercial crops from emerging plant pests and pathogens is growing, due to both increased global trade and a changing environment [1–4]. Britain's trees and woodlands are no exception, with many high-profile issues including acute oak decline (AOD) [5,6], *Hymenoscyphus fraxineus* on ash [7,8], *Phytophthora ramorum* on larch [9,10], oak processionary moth *Thaumetopoea processionea* [11,12] and the Asian long horn beetle *Anoplophora glabripennis* [13]. The combined effect of these pests and diseases is having severe environmental and economic consequences [14–16].

In order to limit the negative impact of pests and disease outbreaks, it is important to quickly understand their distribution and impact, so that control can be implemented in an expedient manner [17,18]. Regulatory surveys to detect and establish the distribution of pests and pathogens are an important first step in this process, but they are resource-intensive and costly [19]. Land managers and regulatory authorities face the prospect of visiting large areas of land in order to detect new threats or monitor existing problems. This is especially true in forested environments, where cropping rotations cover many decades and management is less intensive than in an agricultural or horticultural setting [20]. Detection of new threats is further limited by a complex heterogeneous host landscape, which contains large areas of unmanaged woodland that is hard to access [21,22].

Advances in technology, including smartphone apps for symptom recognition and reporting, have enabled the collection of species distribution data by members of the public to occur with increasing frequency and accuracy [23]. This self-reported information offers the potential to bolster regulatory surveys, which operate with increasingly constrained resources due to the need to respond to a growing list of pests and diseases. However, what self-reported sources tell us about the distribution of pests and diseases is not clear. Detections from members of the public are provided at times and places that suit them, which inherently introduces bias through uneven sampling effort across a landscape [24]. For example, it could be that more positive cases of diseases are reported from areas where there is already heightened awareness, such as around known outbreak foci. However, useful information remains contained within these reports and, given the costs associated with regulatory surveys, it is becoming increasingly necessary to develop methods to work with such data.

Here, a publicly funded survey programme for AOD in England and Wales presents a unique opportunity to ask if data reported from members of the public can be used to accurately estimate distribution of the affected woodland. In 2013, AOD had been documented by landowners in woodlands across large areas of southern England [25]. However, no formal surveys had been conducted, and the prevalence and distribution of AOD was unquantified. AOD affects both species of native oak in Great Britain. *Quercus robur* and *Quercus petraea* represent the largest component of native woodland in Great Britain [26]; as such the potential threat to woodland composition is severe. AOD is part of a wider complex of oak decline agents [27], but has distinctive stem symptoms [5] that enable surveyors to detect potential outbreaks during ground-based surveys [28]. Affected trees are characterized by necrotic lesions in the phloem tissue, which weep dark liquid through cracks in the bark. A suite of necrogenic bacteria are associated with the lesions [6,29] and signs of the native Buprestid beetle *Agrilus bigutttatus* are frequently found on affected trees [30].

Given highly noticeable diagnostic symptoms, a ground-based visual survey is practical for AOD, but represents a costly option due to the large area of host woodland. In England and Wales, there are 192 800 ha of oak scattered among 1 039 000 ha of broadleaf woodland [26]. In order to assess the infection status of a woodland, trained surveyors must travel to each location and spend time searching for symptoms. Clearly, in these situations, a complete census is not possible, and a representative systematic sampling plan is required. By integrating citizen-reported data at the design stage, the systematic survey could maximize the use of available resources and focus on defining the boundaries of the affected area. The survey design implemented in this study created a subset of landowner reports at equal intensity to the survey effort, but this still leaves much valuable information unused. To make use of all data, additional interpolation methods were devolved to predict the affected area and enable samples to be inflated with additional positive detections without a loss of accuracy. This highlights the importance of developing novel strategies tailored to volunteer programmes.

Only visiting a small sample of the potential hosts means that the infection status of most of the woodland is unknown and there is a need to estimate the AOD distribution from a sample. The interpolation procedure developed in this study is based on epidemiological principles, and offers an alternative to geostatistical techniques to estimate the distribution of diseased plants at unsampled locations. Parnell *et al.* [31] and Luo *et al.* [32] developed a method that is suitable for use with records of infection at the level of individual hosts within a field or orchard. By accounting for disease dispersal and spatial structure of host locations, this method accurately predicts infected areas. Here, the use of the method is extended beyond local-scale, host-to-host transmission to predict disease distributions across wider landscapes using grid data, where each cell has an estimate of host abundance.

The objectives of this study are to:

— Assess the accuracy of the stochastic method in relation to geostatistical alternatives across hypothetical surveys with a range of sample sizes.
— Test the effect of using citizen science data, using simulated samples with inflated numbers of positive disease sightings.
— Produce a map showing the estimated distribution AOD in Great Britain.

## 2. Material and methods

### (a) Principles of the survey design

The survey aimed to estimate a national-scale distribution from a limited sample which was constrained by a fixed budget for survey time. To achieve this aim, we investigated the potential to integrate a citizen science dataset collected by Forest Research, which contained landowner reports of AOD-affected sites. As the landowner reports constituted presence-only data, it was unclear how representative they were of the actual AOD distribution. Therefore, a preliminary survey was designed, which evenly spread effort across England and Wales. The survey results were used to test the citizen science data and examine whether AOD was in fact present more widely in the landscape.

A second survey was informed by data from both the landowner reports and the preliminary survey. The design used the principles of risk-based sampling, which have been shown to increase the number of positive detections and reduce survey costs [33]. This study implemented risk-based sampling in the simplest possible manor, by defining a limited survey area which surrounded the known AOD distribution. The survey design proceeded in two stages first selecting 10 km by 10 km squares (hectads) and then identifying individual woods within the selected hectads. Selection of hectads was independent of the existing AOD data, although hectads that were already known to contain AOD-affected woodlands were not resurveyed, but simply retained as a subsample of already positive locations. This reduced the amount of time required on site, effectively giving a larger survey without increasing its cost. The survey design contained hectads that were distributed randomly; however, the subsample had a false-negative rate of zero (there was no chance that symptoms were present and missed during the site visit), so a bias could be present due to imperfect detection during site visits.

To assess the impact of including all the citizen science reports on the predicted AOD distribution, we first needed to develop an analysis method to interpret the data. Once this interpolation method was available, we could assess the impact of including additional landowner reports. The landowner reports only indicated the presence of AOD, so the survey results needed to be simplified to a binary score to be comparable. The impact of using only presence/absence data and including additional citizen science detections was quantified using interpolation methods and test datasets.

The survey design was aided by a collaboration with the Forestry Commission's National Forest Inventory (NFI). This large-scale survey began in 2009 and set out to visit 15 000 (1 ha) plots to assess the UK's woods and forests, although it was only partially through its first cycle in spring 2014 when our survey was designed [26,34]. The use of these sample plots allowed surveyors to target oak woodland and reduce the time spent searching for host trees to survey. This collaboration also enabled the provisional survey findings to be used to estimate a host abundance map for use in the AOD distribution modelling.

Below, we first outline the collection of data on AOD cases from both a large dataset of self-reported cases and from two systematic surveys conducted by Forest Research across England and Wales. We then describe how an estimated map of oak distribution across England and Wales was constructed, before describing a method to estimate the probability of AOD occurrence at unsampled locations. Finally, we test the interpolation methods using data from comprehensive monitoring programmes of disease outbreaks on citrus, which contain complete census information.

### (b) Acute oak decline distribution data

The Forestry Commission operates a Tree Health Diagnostic and Advisory Service (THDAS) that responds to reports sent in by landowners and members of the public. Currently, most new cases arrive via an online tool TreeAlert (http://www.forestry.gov.uk/treealert). On receipt of new reports, causal agents are attributed using descriptions and photographs. For AOD, causal agents are confirmed using real-time PCR protocols. All AOD records from April 2006 were collated from the database (a total of 174 records were received before March 2014 when the main survey was designed).

**Figure 1.**
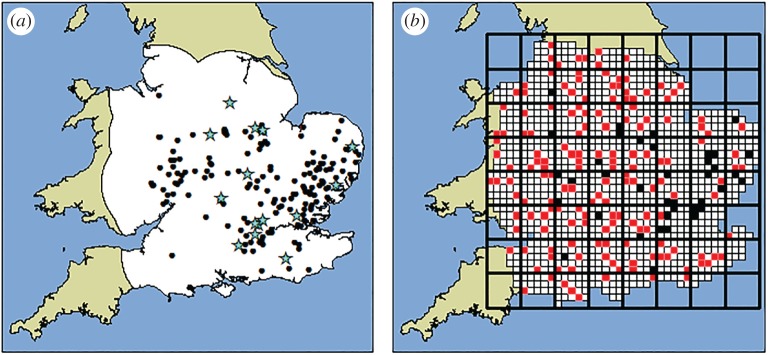
Survey design stages. (*a*) All 191 AOD-positive locations known in 2014, the black dots represent self-reported cases and discoveries made during the preliminary 2013 survey are indicated by stars. The white area shows the buffer region used for survey design (the area of land adjacent to all positive sites, to a distance defined by the maximum nearest neighbour distance between all AOD detections). (*b*) Elements of the survey design. The large squares with thick black lines represent the 50 × 50 km squares. All hectads intersecting the buffer are shown in white, except those selected for survey which are appear filled: the 160 selected for survey shaded in red (grey in greyscale version) and the 38 selections that already contained AOD detections shaded in black. (Online version in colour.)

To make an initial assessment of the quality of THDAS data, a preliminary survey of 116 woodlands was conducted in 2013. This placed equal survey effort across all areas of England and Wales, focusing effort locally in areas of high oak abundance, using coarse 10 × 10 km square (hectad) data from the National Inventory of Woodland and Trees [35]. The preliminary survey detected 17 additional AOD symptomatic sites, all of which were in close proximity to the self-reported cases (figure 1; electronic supplementary material A).

With this in mind, the 2014 survey was designed to improve the definition of the boundaries of the AOD-affected area (figure 1). This was achieved by focusing the survey effort closer to existing sightings and refining survey protocols to increase the number of sites visited. Selection of woodland for survey took place in two stages: first, hectads were selected; second, woodland blocks containing oak were selected at random within the hectad.

An initial step in the design of the 2014 survey was to reduce the area under consideration by defining a buffer region around all known positive sites. The maximum nearest-neighbour distance between any two sites within the 191 known AOD-positive woodlands (approx. 75 km) was used to define a radius around all the positive detections (figure 1*a*). The outermost edge of all the circular areas was used to delimit the extent of the survey area. In this way, the survey design used the distance to the most isolated outbreak and looked the same distance beyond all affected sites. All hectads that intersected the buffer were selected and considered for selection (figure 1*b*). The area was split into (50 × 50 km) squares. These squares were used to stratify random sampling with up to five hectads selected from each. The number of hectads selected in each square was proportional to the land area that fell within the buffer zone, so that approximately a 20% sample was maintained in each square. Stratification spread survey effort more evenly across the area than would have been expected from a simple random selection. Selection considered all hectads that contained Forestry Commission NFI survey sites [26] that contained more than 10% oak area and oak with diameters greater than 15 cm (to exclude recent plantings). If selected squares already contained AOD positives, the locations of these squares were recorded, but they were not surveyed. This created a subsample of THDAS reports that could be used in conjunction with the survey results for analysis. The idealized survey design would have resulted in the selection of 207 hectads; the actual number was slightly lower (198 hectads) due to the available NFI site data in some areas that contained low woodland cover (for example, across the fens in East Anglia). Of the selected hectads, 38 already contained AOD-positive sites and so survey effort was focused in the remaining 160 locations. The inclusion of the AOD-positive subsample increased the percentage of hectads that could be sampled from 13.7 to 17%.

Specific woodlands for survey were selected from the NFI dataset, as these were known to contain oak at the abundance levels described above. Within hectad, a random selection was conducted from all available sites. Where permission could not be gained from the landowners of these sites, replacements were found through collaboration with the woodland trust, national trust, wildlife trusts, forestry commission, local councils and private landowners—this later step was needed in approximately one-third of selected squares in 2013, but only 19% of hectads in 2014. Survey locations were found in 158 squares, as no replacement could be found in the final 2 hectads. AOD survey data were collected between August and September 2014.

### (c) Oak host map

Oak abundance was estimated across the survey area using two data sources: one that recorded the area of broadleaf woodland and a second estimating the proportion of oak within a sample broadleaf woodland. The area of broadleaf woodland was calculated for a 1 km by 1 km grid using shapefiles prepared by the NFI, which record the extent of all woodland (larger than 0.5 ha) across the UK [34]. A second dataset included provisional results from the ongoing NFI survey [26]; this included the abundance of oak within 5476 1 ha survey squares across England and Wales. Data recorded area of native oak species (*Q. robur* and *Q. petraea*) and the total area of broadleaf species within each 1 ha sample square, as estimated by the surveyors. As part of the data-sharing agreement, survey site locations were generalized to give only the hectad that contained the site and required smoothing to fill gaps. The final estimate of oak abundance (hectares of oak) was generated for a smaller grid sizes using the product of the broadleaf area and the proportion of oak within broadleaf area (see electronic supplementary material C for full methods).

### (d) Estimating the distribution of acute oak decline

A statistical method was developed to estimate the distribution of AOD across England and Wales, given the findings of the survey. This used epidemiological principles of dispersal and transmission in conjunction with the estimated host distribution to interpolate between survey points into areas that had not been sampled. The approach builds on a previous method to estimate the intensity of disease across individual host plants [31,32].

The method uses an objective function to estimate the number of infectious agents arriving at a map square, or cell (*Y_i_*), using: the infection state of each of the other cells, *P_j_*; the distance between cells, *d_ij_*; the host abundance for each cell across the landscape; and an exponential dispersal kernel (equation (2.1)). The closer two cells are to each other, the more likely infectious agents will disperse between them. The area of available host within each source cell, Host*_j_*, will influence the number of transmissible units that could disperse from that cell. If there is more host area, there is potentially a bigger outbreak. Finally, the amount of host in the target cell, Host*_i_*, will affect how likely infections are to occur. The interpolation process requires two parameters to be estimated from the data: *α*, the transmission parameter; and *β* the dispersal parameter. *Y_i_* is then transformed to give a probability of host *i* being infected, *P_i_*, using the first term of the Poisson distribution—the probability of no infection given mean *Y_i_* (equation (2.2)).2.1
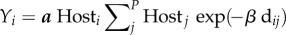
and2.2
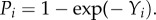


At the outset, infection states are only available for surveyed locations. For all cells without survey data, the probability of infection must be estimated. The probability of infection is updated using a stochastic process that selects maps cells at random before recalculating *P_i_* using equations (2.1) and (2.2). Once a cell is updated, the program checks to see if the change improves the map by assessing how well it fits with results at the surveyed locations. The objective function calculates the expected infection probabilities at each surveyed location, with the absolute difference between the observed and estimated values combined across all survey locations to give the sum of absolute error (SAE). If SAE is reduced, the update is retained in the final map. Cells within the map continue to be selected and updated until SAE stabilizes (when the decrease was less than 0.0001 across the last 5000 cell updates, SAE was judged to be stable and the programme finished). This process is repeated for different values of *α* and *β*, to estimate the optimal parameter values. During this process, each parameter set was repeated to generate 100 realizations of the predicted map, and the average SAE was calculated across realizations and used to compare parameters. The optimal parameter combination was deemed to be the one that resulted in the lowest average SAE, once updates had completed (full methods are described in electronic supplementary material B).

### (e) Testing the interpolation approach and justification for survey design

To fully test the survey methods and interpolation procedures described above, it is necessary to have an accurate dataset that contains all diseased and healthy trees within the landscape. The AOD reports only provide information from a sample of the available woodlands, so an alternative is required. Disease outbreaks on citrus have been the focus of extensive surveys and monitoring efforts, which provide the ideal test datasets. The census data allow for simulated surveys to be designed and conducted, but most importantly enables predicted maps to be directly compared with observed disease records.

Simulated survey locations were generated within the citrus datasets and observed prevalence at these locations was used as a sample for further testing. Using this method, repeated samples could be generated and analysed to assess the quality of predictions. Initially, samples were analysed using both the stochastic method and standard geostatistical techniques to compare the quality of predicted maps. Comparisons were made across a range of sampling intensities (visiting between 4 and 24% of locations containing host trees) using both random selection and stratified approaches.

A second series of tests was conducted to assess how to make the best possible predicted map from a given survey sample. A total of 179 locations were selected using a stratified design that mirrored the AOD survey. Predicted maps were generated for prevalence data and these were compared when only presence/absence information was used for each location. This test is important because landowner reports only indicate disease presence. The impact of including additional landowner reports was assessed by inflating each sample with additional positive detections. The additional ‘landowner’ reports selected either as a random sample of locations with infected trees or as a biased sample where detection was more likely in high-prevalence locations. Both strategies were assessed as two intensities: including either 29 additional infected locations or 145 (1% or 5% of all infected squares, respectively; full methods can be found in electronic supplementary material D).

## 3. Results

### (a) A distribution map for acute oak decline

**Figure 2.**
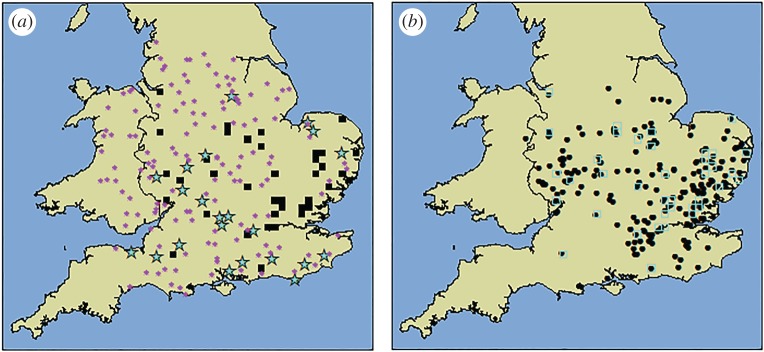
Results from the 2014 survey. (*a*) The locations of all survey sites and selected hectads that already contained AOD reports (black squares). Sites where AOD was detected are shown as turquoise stars and those without symptoms are shown using purple asterisks (grey in greyscale version). (*b*) All self-reported cases used in the final analysis (black dots). The locations of selected squares that were not surveyed are again shown outlined in turquoise (grey in greyscale version). (Online version in colour.)

The 2014 survey resulted in an additional 22 AOD detections (figure 2*a*). In addition, a further 33 self-reported cases arrived before March 2015 and were included in the final dataset for analysis (figure 2*b*). These included three in surveyed hectads where AOD was not detected during the survey. This highlights the issue of imperfect detection; even within selected survey hectads, there remains a large area of woodland that was not surveyed (creating a false-negative rate). The survey process increased awareness of AOD among the local landowners and Forestry Commission staff, and may have exaggerated reporting in these areas, but this demonstrates the added value that citizen scientists can provide.

**Figure 3.**
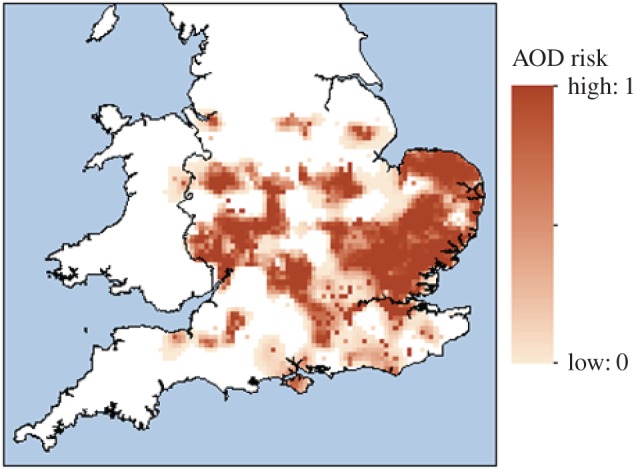
Final prediction of area at risk from AOD. Dark brown areas have a probability of infection of 1 and white areas 0; all shades in between represent intermediate probabilities of infection (with a linear relationship from maximum to minimum; see legend for scale). (Online version in colour.)

The final estimate of the AOD distribution (figure 3) was generated on a 5 × 5 km grid using 246 positive locations (that fell in 208 grid cells) and 137 negative sites (each in a unique cell). In total, 133 of the positive locations were not in survey selected squares, but as landowner reports and survey detections had similar distributions, the data could be combined (see electronic supplementary material A). The final AOD map estimates that 2269 of the 5 × 5 km cells had non-zero values. This equates to an area that is likely to contain AOD-affected woodland of 56 725 km^2^, or approximately 38% of England and Wales.

### (b) Testing the interpolation approach and justification for survey design

The simulated surveys show that the stochastic method gives significantly better predicted maps than kriging (electronic supplementary material D, figure S1). Much of the improvement is due to its ability to define the extent of the infected area, rather than producing a long tail of ever-decreasing probabilities of infection. Unsurprisingly, predicted maps are improved by increased numbers of survey locations: with more data available both methods achieve better estimates. Stratification of survey samples showed no significant improvement in map quality when compared with purely random selections. However, regardless of this finding, stratification was used in the AOD survey design to ensure even coverage.

Predicted maps are most representative of observed epidemics when map cell size is small and not aggregated with neighbours to reduce gaps between surveyed cells. Most importantly, conversion of prevalence to simple presence/absence data was not detrimental to map accuracy; in fact, the latter actually improved the prediction. Finally, the addition of extra positive detections to sparse survey samples (up to 145 additional survey points, which equates to 5% of all infected locations) improved the quality of the predicted maps (electronic supplementary material D, figure S2). In these tests, the overall survey size (179) is comparable with the AOD survey, and the number of additional positive detections is similar to the number of extra locations from landowner reports.

## 4. Discussion

Self-reported data received from landowners and members of the public have enabled a survey for AOD to include more data points than there were resources to visit. The survey method developed in this study involves a simple subsampling approach that can be used to combine pre-existing self-reported data with structured surveys; this targeted official survey effort into areas where new discoveries could be made. This approach offers the potential to improve coverage and/or reduce costs of the surveys in the future. Unstructured data alone have been shown to give poor estimates of population sizes [36], although when datasets are large enough, positive-only reports can be used successfully [37]. For AOD, a survey was designed around the landowner data to add a structure that makes landowner reports useful in later analysis. Similar approaches have been applied to bird surveys in Australia, although these predicted volunteer behaviour and directed official effort away from locations likely to be visited by the public [38]. Previous attempts to use self-reported data to investigate pest and disease problems have either focused on assessing the probability of detection to offset bias [11] or used a structured survey design and directed volunteers to the appropriate locations [23].

The subsampling method alone is wasteful, as it only uses some of the available reports. For AOD, only 27.3% of squares with landowner reports were included in the subsample. By developing an interpolation method in parallel with the survey design, this data loss has been prevented and all the self-reported cases could be used to estimate the AOD distribution. Extensive testing with citrus datasets has shown that samples inflated with additional positive detections produce better quality predicted maps. These results are based on very sparse sampling effort and it should perhaps come as no surprise that additional data points improve predictions. In the case of AOD, analysis was aided by the presence of limited bias in the landowner reports, which shows the importance of effective engagement activities and accessibility of information to the public. Further research is required to understand the potential impact of biased samples on map predictions and to optimize methods for wider application. The latter is likely to require bespoke solutions across a variety of disciplines that aim to interpret spatial distributions. Here, we use epidemiological principles to guide our methods, but similar dispersal processes may influence other spatial associations.

The stochastic method assessed in this paper showed improved predictive power compared with standard geostatistical techniques. These findings at the landscape scale are further supported by previous trials with this technique at the level of individual trees [31,32]. The method is transferable because it relies on general principles and does not require specific knowledge of the system in question. Predicted maps generated from presence/absence data showed similar trends and distributions to those generated with additional prevalence data; in fact, the former had significantly improvements in *κ* scores. Similar trends have previously been shown for species distributions when predictions have been compared using either abundance or occupancy [36]. This finding greatly simplified the process of combining landowner reports with the data collected during the structured survey. The quality of predicted maps was improved by increased sample sizes, with surveys that covered over 20% of the host area most effective. This result is complemented by previous work [31]. However, given a sample of fixed size, maps were most accurate at smaller grid sizes that more closely represented the area covered in the survey sample.

Information gathered by untrained surveyors could be reliably included in the final analysis due to verification processes undertaken by THDAS at Forest Research. Further, the successful discovery of AOD-affected woodland is likely to have been aided by the presence of easy to detect symptoms. Observation skills and expertise of surveyors have been shown to be more important when targets are cryptic or difficult to identify [39]. Untrained surveyors have previously been shown to have provided reports biased towards urban areas [40]. However, this should have been reduced in the case of AOD, due to engagement activities and publications designed to inform landowners and managers who work in woodlands in all situations. Despite this effort, much of the oak grown in Great Britain will not have been visited by those with an awareness of AOD.

The findings of this study suggest that citizen science can be incorporated into survey design successfully. This process is simplified when data reporting takes place without bias across the survey area. Investment in identification guides and engagement with the public will increase awareness and reporting levels. Initial surveys should aim to validate the distribution of citizen reports by visiting locations across the wider landscape. Designed surveys should aim to provide a balanced structure that can incorporate additional citizen science reports. Future studies that aim to understand bias in the distribution of reports could further improve survey design by focusing effort in areas unlikely to be visited by citizen scientists.

## Supplementary Material

Supplementary materials A

## Supplementary Material

Supplementary materials B

## Supplementary Material

Supplementary materials C

## Supplementary Material

Supplementary materials D

## Data accessibility

Data from this study are available in the form of a 5 × 5 km grid showing survey results, this is the resolution used in our final analysis and is sufficient to maintain confidentiality for individual landowners whose woodlands were visited during the survey (http://dx.doi.org/10.5061/dryad.18157 [41]). The National Forest Inventory woodland map is available for download from the Forestry Commission website (https://www.forestry.gov.uk/inventory). The oak abundance data were made available by the NFI for use in this study, but do not belong to the authors. The first phase of the NFI was completed in 2015 and species distribution maps based on the full dataset are currently being prepared for release independently of the authors of this paper.

## References

[1] Brasier CM 2008 The biosecurity threat to the UK and global environment from international trade in plants. Plant Pathol. 57, 792–808. (10.1111/j.1365-3059.2008.01886.x)

[2] Pautasso M, Dehnen-Schmutz K, Holdenrieder O, Pietravalle S, Salama N, Jeger MJ, Lange E, Hehl-Lange S 2010 Plant health and global change—some implications for landscape management. Biol. Rev. 85, 729–755. (10.1111/j.1469-185X.2010.00123.x) 20105153

[3] Wilkinson K et al. 2011 Infectious diseases of animals and plants: an interdisciplinary approach. Phil. Trans. R. Soc. B 366, 1933–1942. (10.1098/rstb.2010.0415) 21624914 PMC3130394

[4] Pautasso M, Schlegel M, Holdenrieder O 2015 Forest health in a changing world. Microb. Ecol. 69, 826–842. (10.1007/s00248-014-0545-8) 25502075

[5] Denman S, Brown N, Kirk S, Jeger M, Webber J 2014 A description of the symptoms of acute oak decline in Britain and a comparative review on causes of similar disorders on oak in Europe. Forestry 87, 535–551. (10.1093/forestry/cpu010)

[6] Denman S, Plummer S, Kirk S, Peace A, McDonald JE 2016 Isolation studies reveal a shift in the cultivable microbiome of oak affected with acute oak decline. Syst. Appl. Microbiol. 39, 484–490. (10.1016/j.syapm.2016.07.002) 27553488

[7] Pautasso M, Aas G, Queloz V, Holdenrieder O 2013 European ash (*Fraxinus excelsior*) dieback—a conservation biology challenge. Biol. Conserv. 158, 37–49. (10.1016/j.biocon.2012.08.026)

[8] Gross A, Holdenrieder O, Pautasso M, Queloz V, Sieber TN 2014 *Hymenoscyphus pseudoalbidus*, the causal agent of European ash dieback. Mol. Plant Pathol. 15, 5–21. (10.1111/mpp.12073) 24118686 PMC6638674

[9] King KM, Harris AR, Webber JF 2015 In planta detection used to define the distribution of the European lineages of *Phytophthora ramorum* on larch (Larix) in the UK. Plant Pathol. 64, 1168–1175. (10.1111/ppa.12345)

[10] Webber JF, Mullett M, Brasier CM 2010 Dieback and mortality of plantation Japanese larch (*Larix kaempferi*) associated with infection by *Phytophthora ramorum*. New Dis. Rep. 22, 19 (10.5197/j.2044-0588.2010.022.019)

[11] Pocock MJO, Roy HE, Fox R, Ellis WN, Botham M 2017 Citizen science and invasive alien species: predicting the detection of the oak processionary moth *Thaumetopoea processionea* by moth recorders. Biol. Conserv. 208, 146–154. (10.1016/j.biocon.2016.04.010)

[12] Williams DT, Straw N, Townsend M, Wilkinson AS, Mullins A 2013 Monitoring oak processionary moth *Thaumetopoea processionea* L. using pheromone traps: the influence of pheromone lure source, trap design and height above the ground on capture rates. Agric. For. Entomol. 15, 126–134. (10.1111/afe.12005)

[13] Straw NA, Fielding NJ, Tilbury C, Williams DT, Inward D 2014 Host plant selection and resource utilisation by Asian longhorn beetle *Anoplophora glabripennis* (Coleoptera: Cerambycidae) in southern England. Forestry 88, 84–95. (10.1093/forestry/cpu037)

[14] Millar CI, Stephenson NL 2015 Temperate forest health in an era of emerging megadisturbance. Science 349, 823–826. (10.1126/science.aaa9933) 26293954

[15] Kautz M, Meddens AJH, Hall RJ, Arneth A 2016 Biotic disturbances in Northern Hemisphere forests—a synthesis of recent data, uncertainties and implications for forest monitoring and modelling. Glob. Ecol. Biogeogr. 26, 533–552. (10.1111/geb.12558)

[16] Freer-Smith PH, Webber JF 2015 Tree pests and diseases: the threat to biodiversity and the delivery of ecosystem services. Biodivers. Conserv. 14, 15-1019 (10.1007/s10531-015-1019-0)

[17] Potter C, Harwood T, Knight J, Tomlinson I 2011 Learning from history, predicting the future: the UK Dutch elm disease outbreak in relation to contemporary tree disease threats. Phil. Trans. R. Soc. B 366, 1966–1974. (10.1098/rstb.2010.0395) 21624917 PMC3130388

[18] Cunniffe NJ, Cobb RC, Meentemeyer RK, Rizzo DM, Gilligan CA 2016 Modeling when, where, and how to manage a forest epidemic, motivated by sudden oak death in California. Proc. Natl Acad. Sci. USA 113, 5640–5645. (10.1073/pnas.1602153113) 27140631 PMC4878485

[19] Parnell S, Gottwald TR, Cunniffe NJ, Alonso Chavez V, van den Bosch F 2015 Early detection surveillance for an emerging plant pathogen: a rule of thumb to predict prevalence at first discovery. Proc. R. Soc. B 282, 20151478 (10.1098/rspb.2015.1478) PMC4571706 26336177

[20] Harmer R, Kerr G, Thompson R 2010 Managing native broadleaved woodland. Edinburgh, UK: TSO.

[21] Mason WL 2007 Changes in the management of British forests between 1945 and 2000 and possible future trends. Ibis (Lond. 1859) 149, 41–52. (10.1111/j.1474-919X.2007.00696.x)

[22] Cunniffe NJ, Koskella BE, Metcalf CJ, Parnell S, Gottwald TR, Gilligan CA 2014 Thirteen challenges in modelling plant diseases. Epidemics 10, 6–10. (10.1016/j.epidem.2014.06.002) 25843374

[23] Meentemeyer RK, Dorning MA, Vogler JB, Schmidt D, Garbelotto M 2015 Citizen science helps predict risk of emerging infectious disease. Front. Ecol. Environ. 13, 189–194. (10.1890/140299)

[24] Isaac NJB, Pocock MJO 2015 Bias and information in biological records. Biol. J. Linn. Soc. 115, 522–531. (10.1111/bij.12532)

[25] Brown N, Inward DJ. G., Jeger M, Denman S 2015 A review of *Agrilus biguttatus* in UK forests and its relationship with acute oak decline. Forestry 88, 53–63. (10.1093/forestry/cpu039)

[26] Brewer A, Ditchburn B 2014 NFI statistical analysis report: 50-year forecast of hardwood timber availability. Edinburgh, UK: Forestry Commission.

[27] Thomas FM 2008 Recent advances in cause–effect research on oak decline in Europe. CAB Rev. 37, 1–12. (10.1079/PAVSNNR20083037)

[28] Brown N, Jeger M, Kirk S, Xu X, Denman S 2016 Spatial and temporal patterns in symptom expression within eight woodlands affected by acute oak decline. For. Ecol. Manage. 360, 97–109. (10.1016/j.foreco.2015.10.026)

[29] Doonan J 2016 Genomic analysis of bacterial species associated with acute oak decline. PhD thesis, School of Biological Sciences, Bangor University, UK.

[30] Brown N, Jeger M, Kirk S, Williams D, Xu X, Pautasso M, Denman S 2017 Acute oak decline and *Agrilus biguttatus*: the co-occurrence of stem bleeding and D-shaped emergence holes in Great Britain. Forests 8, 87 (10.3390/f8030087)

[31] Parnell S, Gottwald TR, Irey MS, Luo W, van den Bosch F 2011 A stochastic optimization method to estimate the spatial distribution of a pathogen from a sample. Phytopathology 101, 1184–1190. (10.1094/PHYTO-11-10-0311) 21916625

[32] Luo W, Pietravalle S, Parnell S, Van den Bosch F, Gottwald TR, Irey MS, Parker SR 2012 An improved regulatory sampling method for mapping and representing plant disease from a limited number of samples. Epidemics 4, 68–77. (10.1016/j.epidem.2012.02.001) 22664065

[33] Parnell S, Gottwald TR, Riley T, Van Den Bosch F 2014 A generic risk-based surveying method for invading plant pathogens. Ecol. Appl. 24, 779–790. (10.1890/13-0704.1) 24988776

[34] Forestry Commission. 2011 National forest inventory report: NFI 2011 woodland map GB. Edinburgh, UK: Forestry Commission.

[35] Forestry Commission. 2003 National Inventory of woodlands and trees: Great Britain. Edinburgh, UK: Forestry Commission.

[36] Kamp J, Oppel S, Heldbjerg H, Nyegaard T, Donald PF 2016 Unstructured citizen science data fail to detect long-term population declines of common birds in Denmark. Divers. Distrib. 22, 1024–1035. (10.1111/ddi.12463)

[37] Woodcock BA, Isaac NJB, Bullock JM, Roy DB, Garthwaite DG, Crowe A, Pywell RF 2016 Impacts of neonicotinoid use on long-term population changes in wild bees in England. Nat. Commun. 7, 12459 (10.1038/ncomms12459) 27529661 PMC4990702

[38] Tulloch AIT, Mustin K, Possingham HP, Szabo JK, Wilson KA 2013 To boldly go where no volunteer has gone before: predicting volunteer activity to prioritize surveys at the landscape scale. Divers. Distrib. 19, 465–480. (10.1111/j.1472-4642.2012.00947.x)

[39] Kelling S, Johnston A, Hochachka W, Iliff M, Fink D 2015 Can observation skills of citizen scientists be estimated using species accumulation curves? PLoS ONE 10, e0139600 (10.1371/journal.pone.0139600) 26451728 PMC4599805

[40] Geldmann J, Heilmann-Clausen J, Holm TE, Levinsky I, Markussen B, Olsen K, Rahbek C, Tottrup AP, Leung B 2016 What determines spatial bias in citizen science? Exploring four recording schemes with different proficiency requirements. Divers. Distrib. 22, 1139–1149. (10.1111/ddi.12477)

[41] Brown N, van den Bosch F, Parnell S, Denman S 2017 Integrating regulatory surveys and citizen science to map outbreaks of forest diseases: acute oak decline in England and Wales. *Dryad Digital Repository*. (10.5061/dryad.18157) PMC5543216 28724732

