# Supplementary material for: Integrating regulatory surveys and citizen science to map outbreaks of forest diseases: acute oak decline in England and Wales
Source: Proc Biol Sci. 2017 Jul 19;284(1859):20170547. doi: 10.1098/rspb.2017.0547 (PMC5543216; doi:10.1098/rspb.2017.0547)
Supplement: Supplementary materials A [file rspb20170547supp1.docx]

Supplementary material A

2013 survey


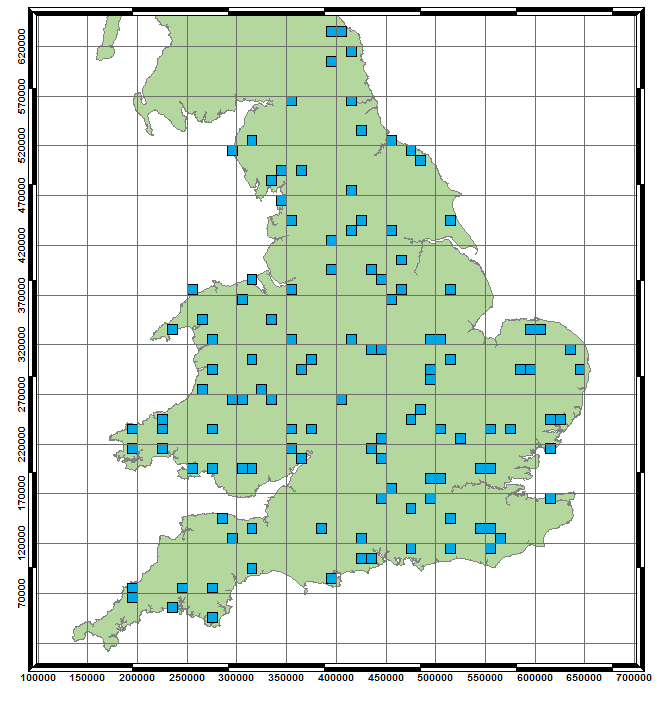


Figure 1: Hectads selected for the 2013 Survey

Association between survey detections and landowner reports

This association holds across both surveys, with the landowner reports clustering around the combined survey detections (figure 2). A variant of Ripley’s k, the L function was used to assess the co-occurrence of detections across a range of distances using the R package spatstat. The two detection regimes did not however produce patterns of the same intensity (Figure 3), with landowner reports showing signs of local clustering between 30 and 70 km. This could be representative of areas of high disease prevalence or indicative of local bias.


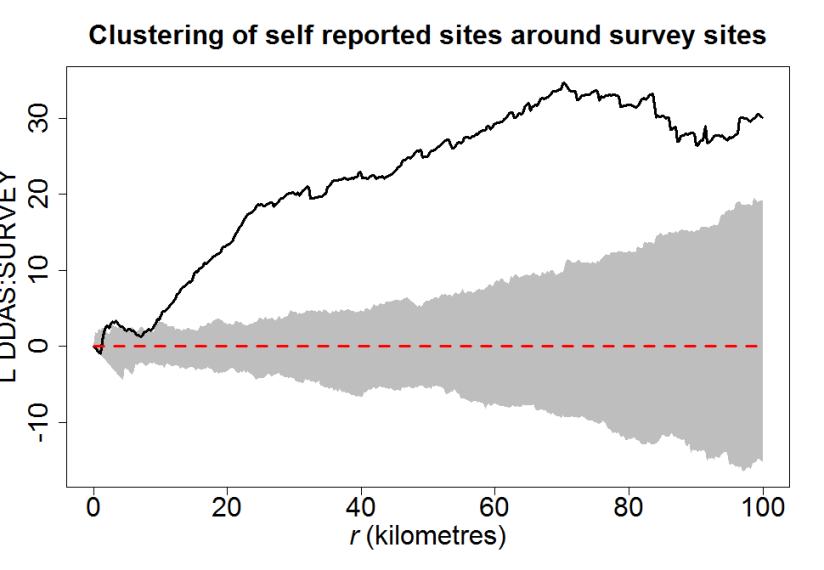


Figure 2: Clustering of landowner reports around all positive detections from the 2013 and 2014 surveys. The grey shaded area shows expected association if landowner detections were independently distributed (based on 99 random point pattern simulations).


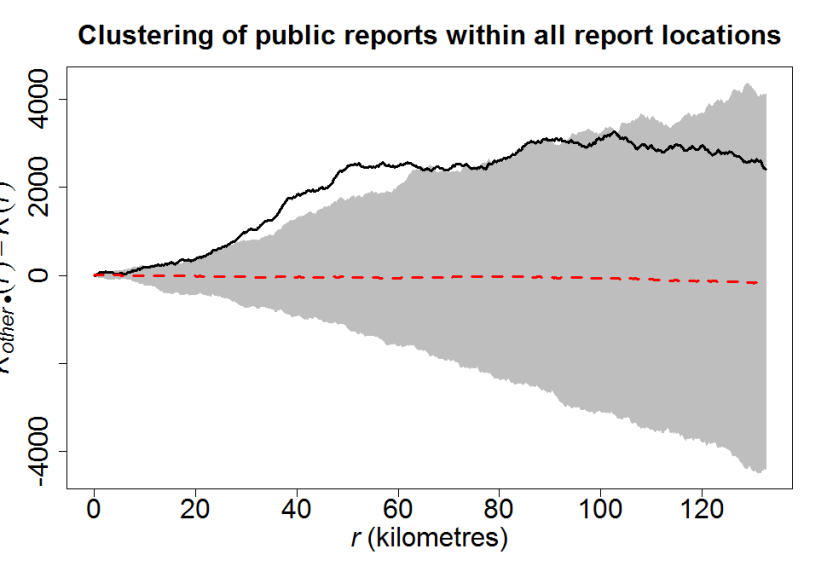


Figure 3: Clustering of landowner reports within the locations of all positive detections. The grey shaded area shows expected association if both methods were independently distributed (based on 99 simulations, which randomly assigned labels across the observed locations).
