# Supplementary material for: Integrating regulatory surveys and citizen science to map outbreaks of forest diseases: acute oak decline in England and Wales
Source: Proc Biol Sci. 2017 Jul 19;284(1859):20170547. doi: 10.1098/rspb.2017.0547 (PMC5543216; doi:10.1098/rspb.2017.0547)
Supplement: Supplementary materials B [file rspb20170547supp2.docx]

Supplementary material B

# Estimating a distribution from a sample

A statistical method was developed to estimate the distribution of AOD across England and Wales given the findings of the survey. This used epidemiological principles of dispersal and transmission in conjunction with the estimated host distribution to interpolate between survey points into areas that had not been sampled. The approach builds on a previous method to estimate the intensity of disease across individual host plants [31,32].

The method uses an objective function to estimate the number of infectious agents arriving at a map square, or cell, (Y*_i_*) using: the infection state of each of the other cells, P_j_; the distance between cells, d_ij_; the host abundance for each cell across the landscape; and an exponential dispersal kernel (1). The closer two cells are to each other the more likely infectious agents will disperse between them. The area of available host within each source cell,${Host}_{j}$, will influence the number of transmissible units that could disperse from that cell. If there is more host area, there is potentially a bigger outbreak. Finally, the amount of host in the target cell, ${Host}_{i}$, will affect how likely infections are to occur. The interpolation process requires two parameters to be estimated from the data; α, the transmission parameter, and β the dispersal parameter. Y_i_ is then transformed to give a probability of host *i* being infected, P_i_, using the first term of the Poisson distribution – the probability of no infection given mean Y_i_ (2).

$Y_{i}= \boldsymbol{a}{Host}_{i}\sum P_{j}{{Host}_{j}\exp\left( - \beta d_{ij} \right)}$ (1)

$P_{i}=1-exp(-Y_{i})$ (2)

At the outset, infection states are only available for surveyed locations, as their status was observed. For all cells without survey data the probability of infection must be estimated. The probability of infection is updated using a stochastic process that selects maps cells at random before recalculating P_i_ using equations (1) and (2). Once a cell is updated the program checks to see if the change improves the map by assessing how well it fits with results at the surveyed locations. The objective function calculates the expected infection probabilities at each surveyed location, with the absolute difference between the observed and estimated values combined across all survey locations to give the sum of absolute error (SAE). If SAE is reduced the update is retained in the final map. Cells within the map continue to be selected and updated until SAE stabilises (when the decrease was less than 0.0001 across the last 5000 cell updates SAE was judged to be stable and the program finished). This process is repeated for different values of α and β, to estimate the optimal parameter values. During this process, each parameter set was repeated to generate 100 realisations of the predicted map, the average SAE was calculated across realisations and used to compare parameters. The optimal parameter combination was deemed to be the one that resulted in the lowest average SAE once updates had completed (full methods are described in supplementary materials B).

At the outset, infection states are only available for surveyed locations, as their status was observed. The method can either estimate for presence/ absence data (0 or 1), or where disease prevalence is known (the proportion of infected hosts in the sample). The effect of differing starting conditions was assessed (see below). For all cells without survey data the probability of infection must be estimated. The probability of infection is subsequently calculated for all cells in the landscape, through a stochastic update process. For prevalence data, the method estimates the probability of a tree within the square being infected whereas for presence / absence data the probability relates to the chance of infection occurring within the cells. To begin this process, locations that had not been sampled were assumed to have no infection. Cells are then selected at random and their probability of infection is calculated using the objective function. Once a new probability of infection has been calculated the change is assessed to see if it improves the predicted map. The quality of the predicted map is quantified by comparing its fit to the observed survey results. The fit of the map is assessed using the objective function value at each survey site locations, this gives an expected infection probability. The absolute difference between the observed and sum of expected infection probabilities calculated across all survey site locations to give the sum of absolute error (SAE) across the predicted map. SAE is calculated first for the initial map containing only infections at survey sites, and then recalculated following the updated estimate for each randomly selected cell. When updated cell estimates reduce SAE they are retained to improve the predicted map, and the new SAE value is used for future comparisons. The stochastic process continues, selecting and updating additional cells, until the SAE stabilises and a “best fit” map is produced. The progress of the objective function was monitored using the change in SAE after every 100 updates, and when the decrease was less than 0.0001 across the last 5000 cell updates SAE was judged to be stable and the program finished. A final measure of SAE is made using the average outputs of multiple runs to give a stable state estimate for the parameter set in question.

This process is repeated for different combinations of α and β, for which optimal parameter values were estimated. This was achieved using a phased grid search: At the outset a reasonable range for each parameter was decided (α 0-20, β 2-10), the program was then run for all parameter sets within these ranges, at a course resolution with 0.5 increments. This resulted in course grid of estimates which could be used to generate a heat map. The parameter set with the minimum SAE value was used to centre a finer scale grid search covering half the range of both α and β in increments of 0.1. During this process, each parameter set was repeated to generate 100 realisations of the predicted map, the average SAE was calculated across realisations and used to compare parameters. The final prediction of infection probability was produced using the parameter set that gave the minimum SAE value, and generated a map using the average cell value (P*_i_*) from 1000 runs of the program. Dispersal parameters are estimated using a single snapshot provided by the survey data and as such are not predictive of future expansion.

Computational efficiencies for the stochastic method:

Because of the need to repeat the map calculations multiple times for each parameter set and test many combinations of parameters the program becomes computationally expensive, so efforts were made to optimise the algorithms for time savings. Due to the regular pattern of the cells, which formed a regular grid, efficiency savings can be made by using a pre-calculated distance matrix. This stored the distances between the centre of the target cell and the centres of adjacent cells across the landscape. Further savings were made by limiting the size of the kernel, and thus the distance over which the objective function calculated potential dispersal, so that it was limited to cells that fall closer to the target cell. At large distances donor cells only contribute smaller amounts to Y*_i_* and have negligible effects on the estimated infection probability (P*_i_*) (Luo, 2012). The inclusion, or exclusion, of cells within the kernel is decided based on their maximum possible contribution to Y*_i_*, with a threshold set at 0.00001. The threshold distance, was calculated for each parameter set using equation (4), and assumed the maximum contribution possible from P*_j_* and maximum host abundance in both target and donor cells.

$d_{ij}\leq-{\ln\left( \frac{\Delta Y_{i}}{a\left( {Host}_{max} \right)^{2}} \right)}/\beta$ (4)

where P*_j_* = 1.

Due to the use of a kernel that limited the number of adjacent cells included when calculating the objective function, further efficiency savings could be be made when calculating SAE and assessing the fit of the map. After the randomly selected cell has been updated, absolute error will only change at survey points that fall within distance d*_ij_* of the selected cell. The objective function is only used once to update the selected cell, but then the same calculation is required for each survey point (of which there are potentially many hundreds, or more, across the landscape) in the calculation of SAE. This step massively reduces the time taken between cell updates.

Following initial trials of the method host contribution was modified to use relative abundance by dividing by area of host plants, or the number of host plants, in each cell by the maximum value in the landscape. This increased the consistency of parameter estimations across different disease maps.
