# Supplementary material for: Integrating regulatory surveys and citizen science to map outbreaks of forest diseases: acute oak decline in England and Wales
Source: Proc Biol Sci. 2017 Jul 19;284(1859):20170547. doi: 10.1098/rspb.2017.0547 (PMC5543216; doi:10.1098/rspb.2017.0547)
Supplement: Supplementary materials C [file rspb20170547supp3.docx]

Supplementary material C

Oak host map

Oak abundance was estimated across the survey area using two data sources: one estimating the area of broadleaf woodland; and a second estimating the proportion of oak within broadleaf woodland. Firstly, the area of broadleaf woodland was calculated using shapefiles prepared by the NFI, these weredigitised from aerial photography and show the extent of all woodland greater than 0.5 ha [34]. Here woodland was split into five categories (Broadleaf composition of each is shown in parentheses): Broadleaf (90%); Mixed mostly broadleaf (65%); Mixed mostly conifer (35%); Conifer (10%); Coppice with standards (90%). The area of each category was calculated within each cell of a 1 km x 1 km grid, and then multiplied by its respective broadleaf proportion to give a final broadleaf area estimate for each cell. A second dataset was made available by the NFI, this included provisional results from their survey[26] this included the proportion of oak within 5,476 one hectare survey squares across England and Wales. As part of the data sharing agreement survey site locations were generalised to give only the hectad that contained the site. Data recorded area of native oak species (*Q. robur* and *Q. petraea*) and the total area of broadleaf species within each 1 ha sample square, as estimated by the surveyors. The NFI survey allocated survey effort in proportion to total woodland area and as such some cells had little, or no, broadleaf information. To overcome this issue the data were adaptively smoothed, so that each cell estimate was based on a minimum of 4 ha of broadleaf woodland. Where the estimate could not be generated from the available data within a hectad then the sample area was increased to include adjacent hectads . The adaptive smoothing process progressed through multiple iterations, each increasing the radial distance of the smoothing kernel, until the sample contained 4 ha of broadleaf woodland cover. This ensured that the sample always contained information from the minimum number of adjacent cells needed to meet the 4 ha criteria. The final estimate of oak abundance (hectares of oak) was generated for a smaller grid sizes using the product of the broadleaf area and the proportion of oak within broadleaf area. Woodland areas were calculated using ArcGIS (ESRI), and the adaptive smoothing conducted using a program in C.


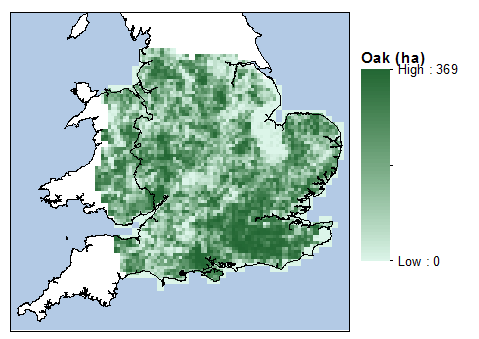


Figure 1: Estimated abundance of oak across the buffer region. Estimates were generated for a 5 km x 5 km grid using preliminary NFI survey data and 2013 woodland maps. NFI survey data was provided at 10km resolution and smoothed to ensure all hectads contained an estimate.
