# Supplementary material for: Integrating regulatory surveys and citizen science to map outbreaks of forest diseases: acute oak decline in England and Wales
Source: Proc Biol Sci. 2017 Jul 19;284(1859):20170547. doi: 10.1098/rspb.2017.0547 (PMC5543216; doi:10.1098/rspb.2017.0547)
Supplement: Supplementary materials D [file rspb20170547supp4.docx]

Supplementary material D

# Testing the stochastic method

# Methods

Validation of the method:

The accuracy of the method could be fully tested using two additional datasets collected in Florida: one recording a Huanglongbing (HLB) outbreak in a plantation setting [1–3]; and one recording citrus canker in Miami [4,5]. In both cases the surveys recorded a complete census of all potential hosts within the site boundaries. The 8.4 km^2^ citrus plantation monitored for HLB contained over 250,000 individual trees (sweet orange, *Citrus sinesis* (L.)), planted in 180 blocks. The development of the HLB epidemic was monitored between November 2005 and July 2007, analyses in this paper use data from the second round of survey. Citrus canker data are used from site D2, this 5.2 km^2^ area of urban Miami contained approximately 6,000 citrus trees (mixed species), irregularly scattered within gardens. At this location monitoring was conducted between October 1997 and November 1999, analyses in this paper use the final distribution of diseased trees.

A gridded map was generated from each dataset, by first dividing the area into equal sized quadrates then calculating the number of host trees in each, as well as the proportion that were infected. For Southern Gardens each quadrat represented a 1 ha square and for Miami the quadrats were smaller, 50 m x 50 m, so that the total number of cells in the final map were comparable (Southern Gardens: 900 cells, 890 containing host plants. Miami: 1575 cells, 919 containing host plants).

To assess the accuracy of the method, sub samples were selected from the gridded map and treated as the results of a survey. Predicted maps were then estimated from the sub-sample and directly compared to the observed census data.

Test 1: Optimum sample size and strategy

This test compares the accuracy of the method with kriging. Assessments are conducted across a range of sampling intensities (the percentage of possible cells that were included in the sub-sample) and compare to selection strategies: A purely random selection and a stratified approach that matches with that used for the AOD survey. Ten selections were made using each method at all of the following sampling intensities tested: 4%, 8%, 12%, 16%, 20%, and 24% of the surveyed area. The stratified sample firstly divided each host map into large stratification squares (5 x 5 cells), with random samples then made within each square. For strata that lacked hosts in each cell sampling effort was adjusted to ensure the number of samples was proportional to host coverage. The stratified sample mirrored the AOD survey design.

In order to assess the relative accuracy of predictions made by the stochastic method each sample of survey sites was also interpolated using kriging. Kriging quantifies the amount of variation between samples at different spatial distances, and then fits a variogram (curve) to these data. The variogram can then be used to predict values at unsampled locations. Kriging was conducted in R using the package GeoR. An exponential variogram shape was used with REML parameter estimation [6,7].

Test 2: Maximising map accuracy

1. What scale gives the best predicted map from existing survey data?

This test compared maps generated at different scales from the same 30 sub-samples. This test used only the Southern Gardens data, due to the high number and density of surveyed trees. Sub-samples were generated from a fine scale gridded map, with 25 m X 25 m cells. Selection of sub-samples took place using a stratified method, where, 1 ha squares were first selected as above. Then in an additional step a single 25 m cell was selected within the 1 ha square to mirror the AOD survey which monitored an individual wood with each hectad. Survey results were aggregated to generate predicted maps at three scales (with 25 m, 50m and 100 m edged squares). Due to the two phased selection procedure no two survey points could fall within the larger sized squares.

1. Prevalence Vs. Presence/Absence

This test compared the methods predictions with prevalence data to those with only presence / absence information. The same survey samples generated above were converted to binary presence / absence (1 or 0) format and so that the accuracy of the methods predictions could be compared directly with surveys containing prevalence data. All surveys were again aggregated across the same three scale grids.

1. Incorporating extra disease detections

This test assessed the impact of surveys where the number of positive sightings was inflated. Allowing an assessment of the impact of including all THDAS data alongside the survey selected squares. This test used the same presence / absence selections used above, but added additional positive squares to each. Additional positives were added in two ways, either following a purely random selection across the survey area or with a selection bias toward high prevalence cells. For the later, cells were selected at random, but only included in the sub-sample when a second random number was smaller than the cells prevalence. Both selections were repeated to include the addition of 1 % of all infected squares and 5 % of all infected squares. All surveys were again aggregated across the same three scale grids.

Tests of map accuracy.

Predicted maps were compared to the observed data using two methods: firstly, the Kappa statistic [8] assessed how well cells were correctly assigned as healthy and diseased; and secondly, the mean absolute error (AE) was calculated, using the sum of the absolute difference between predicted and observed infection probabilities for all cells, divided by the total number of cells. The mean AE was used to compare how accurately predictions correspond to observed values, with smaller values indicating a better match. The Kappa statistic gives a measure of the proportion of correctly predicted diseased and healthy cells, once the probability of chance agreement has been removed (5) (6) (Cohen, 1960; Madden *et al*., 2007). Kappa (K) is generated using a four element matrix that contains the number of: true positives (tp), true negatives (tn), false positives (fp) and false negatives (fn) across the map.

$K=\frac{\left( tp-tn \right)-C}{n-C}$ (5)

Where n is the total number of cells in the map

$C= \frac{\left( tp+fn \right)\left( tp+fp \right)+ \left( tn+fn \right)\left( tn+fp \right)}{n}$ (6)

If the predicted map is only as accurate as a randomly generated map (based on the mean prevalence of disease in the sample) kappa will return a value of zero. A score of one would indicate a perfect match between the observed and predicted maps. The estimated probabilities in the predicted map were converted to binary responses using a threshold based on the average estimated probability across the map; above which cells were deemed infected (Liu *et al*., 2005; Parnell *et al*., 2011).

Statistical analysis was completed using R 3.1.2 and the package multicomp was used for post-hoc significance testing. For test 1: analysis of variance (anova) was conducted using Kappa scores, the effects of mapping method, sample size and selection method were used in the model with additional error terms to account for site and sample within site (the same data was used for kriging and the stochastic method). Anova was also conducted for TAE. The model included the same factors as above, but each site was assessed separately, to improve model fit. For test 2: anova was again conducted using Kappa scores, the effects of survey selection type and final map scale (grid cell size). Additional error terms were used to control for error caused by re-using the original 20 selections, and also by map scale within selection (the same 20 samples were used repeatedly and these selections caused consistent aggregated patterns at larger map cell sizes). Model checking (residual vs fitted values and QQ plots) and post-hoc Tukey tests we completed using anova models without the additional split plot error structures.

Results

Test 1: Optimum sample size and strategy

Maps produced with the stochastic method had significantly higher Kappa scores (F_1,464_ = 4142.42, *p* < 0.001) than those produced with Kriging (Figure 1) and the Kappa varied significantly between sample sizes (F_5,464_ = 26.44, *p* < 0.001). Tukey tests show that a 4 percent selection had significantly lower kappa scores than 16, 20 and 24 percent selections (t = 3.13, *p* = 0.02; t=3.84, *p* = 0.002; and t = 5.05, *p* < 0.001 respectively). In addition, 24 percent samples had significantly higher kappa scores than 8 and 12 percent selections (t = 3.23, *p* = 0.016; and t = 3.6, *p* = 0.005 respectively). There was also a significant interaction between analysis method and sample percentage with the difference in Kappa scores increasing as selection percentage increased (F_5,464_ = 3.42, *p* = 0.005). Finally, there was no significant difference between random selections and stratified samples (F_1,464_ = 0.029, *p* =0.87).

Mean AE was significantly lower when the stochastic method was used (SG F_1,231_ = 50.88, *p* < 0.001; Miami F_1,231_ = 10.88, *p* = 0.001), as such maps were more representative of the actual epidemic. Sample size had a significant effect on mean AE (SG F_5,231_ = 113.64, *p* < 0.001; Miami F_5,231_ = 30.44, *p* < 0.001), with larger sample sizes reducing error in the predicted map. Selection method had no effect on mean AE (SG F_1,231_ = 2.44, *p* = 0.12; Miami F_1,231_ = 0.4, *p* < 0.85).


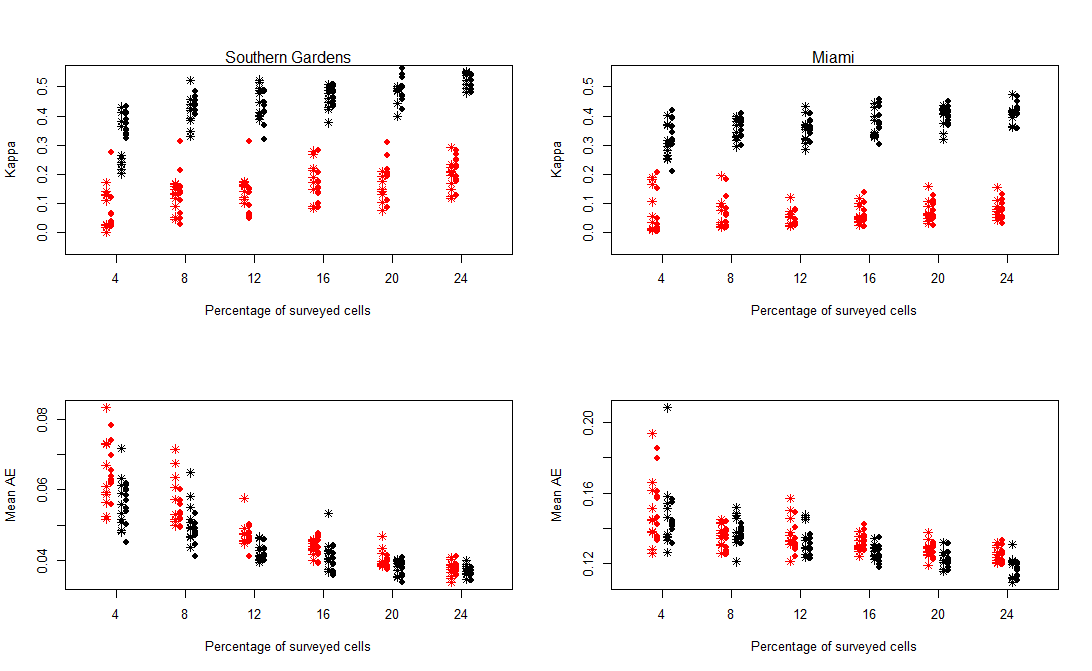


Figure 1: Comparison of stochastic method with kriging. All black points summarise maps generated using the stochastic method and all red points summarise maps produced using kriging. Results for Southern Gardens are shown in the left two panels and results for Miami are shown in the panels on the right. Kappa statistics are shown in the top panels and mean absolute error is shown in the lower panels. Finally, filled circles represent survey stratified selections and asterisks represent random samples.

Test 2: Maximising the predictive value of risk maps

There was a significant difference in kappa scores between maps produced on different scale grids (Figure 2; F_2,58_ = 239.8 *p* < 0.001). Tukey tests show that a 50 m grid is most accurate, kappa is significantly greater than for a 25 m grid (t = 5.8, *p* < 0.001) and a 100 m grid is the most innaccurate, it has a lower kappa than either a 25 m grid (t=9.04, *p* < 0.001) or a 50 grid (t = 14.84 , *p* <0.001).

Across the different types of survey selection tyes there were significant differences (F_5, 435_ = 576.17 *p* <0.001). Suprisingly samples with only Yes/No information had higher kappa scores than when prevalence data was available (t =3.99 *p* = 0.001). All methods and intensities of inflating the sample with additional positive sightings significantly increased kappa compared to the original sample alone (Random 1% t =7.39 *p* < 0.001; Random 5% t = 7.54 *p* < 0.001; prevalence dependent detection 1 % t = 7.36 *p* < 0.001; and prevalence dependent detection 5%, t = 10.0 *p* < 0.001), although there was no significant difference between the different types of inflated samples themselves. The Interaction between survey type and map scale was also significant (F_10,435_ =28.13 *p* < 0.001), although this explained much less variation than the main effects themselves and is due to differneces between the selection types at the 100 m grid size.


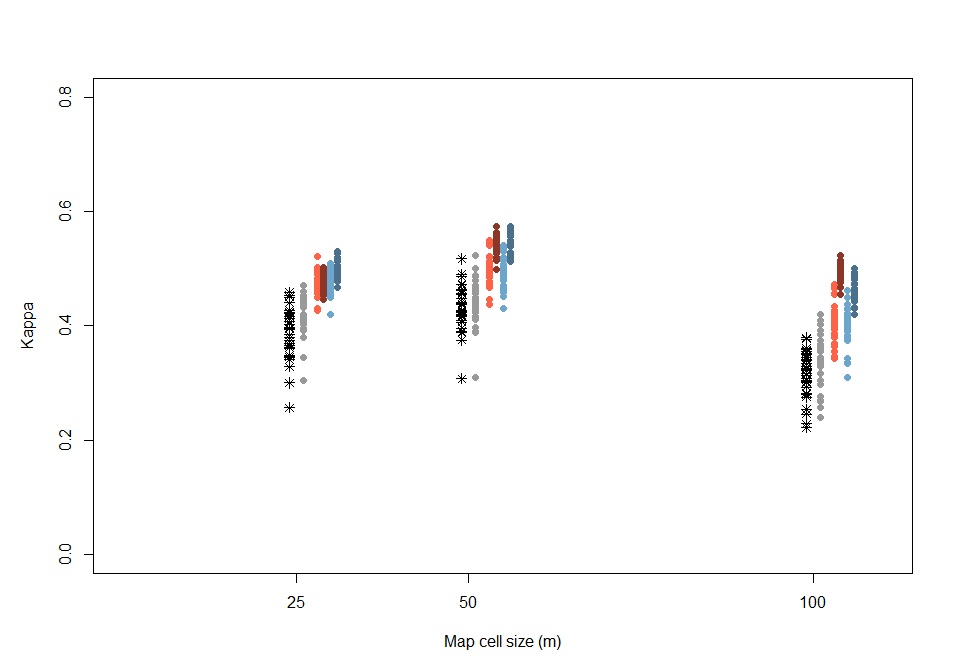


Figure 2: Kappa statistics from trials to maximise the predictive values of risk maps. Black asterisks shown scores from maps generated with prevalence data; grey circles show results with presence absence data; light red dots show results from surveys randomly inflated with extra positive detections, from 1 % of infected squares; dark red dots represent a 5 % random inflation of positive detections; Light blue represents an additional 1 % inflation with prevalence dependent detections; and dark blue a 5 % inflation with prevalence dependent detections. Results are shown for the three map scales.

# References

1. Parry, M., Gibson, G. J., Parnell, S., Gottwald, T. R., Irey, M. S., Gast, T. C. & Gilligan, C. a 2014 Bayesian inference for an emerging arboreal epidemic in the presence of control. *Proc. Natl. Acad. Sci. U. S. A.* **111**, 6258–62. (doi:10.1073/pnas.1310997111)

2. Gottwald, T. R. 2010 Current Epidemiological Understanding on Citrus Huanglongbing. *Annu. Rev. Phytopathol.* **48**, 119–139. (doi:10.1146/annurev-phyto-073009)

3. Parnell, S., Gottwald, T. R., Irey, M. S., Luo, W. & van den Bosch, F. 2011 A stochastic optimization method to estimate the spatial distribution of a pathogen from a sample. *Phytopathology* **101**, 1184–1190.

4. Gottwald, T. R., Sun, X., Riley, T., Graham, J. H., Ferrandino, F. & Taylor, E. L. 2002 Geo-referenced spatiotemporal analysis of the urban citrus canker epidemic in Florida. *Phytopathology* **92**, 361–377. (doi:10.1094/PHYTO.2002.92.4.361)

5. Parnell, S., Gottwald, T. R., Cunniffe, N. J., Alonso Chavez, V. & van den Bosch, F. 2015 Early detection surveillance for an emerging plant pathogen: a rule of thumb to predict prevalence at first discovery. *Proc Biol Sci R Soc* **282**, 20151478-. (doi:10.1098/rspb.2015.1478)

6. Ribeiro jr., P. J. & Diggle, P. J. 2001 geoR: A package for geostatistical analysis. *R-News* **1**, 15–18. (doi:10.1159/000323281)

7. Oliver, M. A. & Webster, R. 2014 A tutorial guide to geostatistics: Computing and modelling variograms and kriging. *Catena* **113**, 56–69. (doi:10.1016/j.catena.2013.09.006)

8. Madden, L., Hughes, G. & van den Bosch, F. 2007 *The study of plant disease epidemics*. St Paul, Minnesota: APS Press.
